# The Impact of Geographical Variation in *Plasmodium knowlesi* Apical Membrane Protein 1 (PkAMA-1) on Invasion Dynamics of *P. knowlesi*

**DOI:** 10.3390/tropicalmed8010056

**Published:** 2023-01-10

**Authors:** Yee Ling Ng, Wenn-Chyau Lee, Yee-Ling Lau, Mun Yik Fong

**Affiliations:** 1 Department of Parasitology, Faculty of Medicine, Universiti Malaya, Kuala Lumpur 50603, Malaysia; 2 A*STAR Infectious Diseases Labs, Agency for Science, Technology and Research (A*STAR), Singapore 138648, Singapore

**Keywords:** *Plasmodium knowlesi*, PkAMA-1, merozoite invasion, Peninsular Malaysia, Malaysian Borneo

## Abstract

*Plasmodium knowlesi* has emerged as an important zoonotic parasite that causes persistent symptomatic malaria in humans. The signs and symptoms of malaria are attributed to the blood stages of the parasites, which start from the invasion of erythrocytes by the blood stage merozoites. The apical membrane protein 1 (AMA-1) plays an important role in the invasion. In this study, we constructed and expressed recombinant PkAMA-1 domain II (PkAMA-1-DII) representing the predominant haplotypes from Peninsular Malaysia and Malaysian Borneo and raised specific antibodies against the recombinant proteins in rabbits. Despite the minor amino acid sequence variation, antibodies raised against haplotypes from Peninsular Malaysia and Malaysian Borneo demonstrated different invasion inhibition (46.81% and 39.45%, respectively) to *P. knowlesi* A1-H.1, a reference strain derived from Peninsular Malaysia. Here, we demonstrated how a minor variation in a conserved parasite protein could cast a significant impact on parasite invasion biology, suggesting a complex host-switching of *P. knowlesi* from different locations. This may challenge the implementation of a standardized One Health approach against the transmission of knowlesi malaria.

## 1. Introduction

*Plasmodium knowlesi* is an emerging zoonotic parasite transmitted by several anopheline mosquitoes with simio-anthropophilic biting behavior [1]. The persistent transmission of knowlesi malaria from natural macaque hosts to humans has challenged the malaria elimination efforts in Southeast Asia, particularly in Malaysia [2]. Controlling knowlesi malaria requires a One Health approach that focuses on the human-animal-environment interface sustaining the disease transmission. To date, most of the malaria cases reported in Malaysia were attributed to *P. knowlesi*, with 87% of the cases being reported from Malaysian Borneo [3]. Apart from the diagnostic challenges to differentiate *P. knowlesi* from other species of medically important malaria parasites via light microscopy (due to shared morphological features of the parasite blood stages) [4,5,6], zoonotic malaria caused by *P. knowlesi* is potentially fatal, with severe complications manifest in the forms of acute kidney injury (AKI), acute respiratory distress syndrome (ARDS), jaundice, and severe anemia [7,8,9,10,11]. This simian malaria parasite demonstrates high invasion plasticity, where erythrocytes derived from both humans and indigenous simians are susceptible to *P. knowlesi* [12,13,14,15,16,17]. The invasion of *Plasmodium* blood-stage merozoite is driven by several key components, including apical membrane protein 1 (AMA-1) [18,19,20,21,22,23]. In fact, PkAMA-1 has gained research attention in studies associated with parasite invasion biology [24,25,26]. By using rat monoclonal antibody raised against the invasion-inhibitory epitope of PkAMA-1, Mitchell et al. elegantly demonstrated the critical role of PkAMA-1 in reorientating the merozoite after the initial attachment of the merozoite to the erythrocyte surface [27]. Recently, we investigated the genetic diversity of *PkAMA-1* among *P. knowlesi* clinical isolates from Malaysia and unraveled haplotypes that were distinct to Peninsular Malaysia and Malaysian Borneo, despite the relatively conserved nature of this gene [28]. Interestingly, each geographical area is represented by a predominant haplotype [27,28]. Nevertheless, the impact of these distinct PkAMA-1 geographical haplotypes on the invasion dynamics of *P. knowlesi* remained unknown. In this study, we expressed recombinant proteins of predominant haplotypes of PkAMA-1 domain II from Peninsular Malaysia and Malaysian Borneo, synthesized antibodies against the recombinant proteins, and evaluated the effect of respective antibodies on the blood stage merozoite invasion efficacy of *P. knowlesi* A1-H.1, a reference strain derived from Peninsular Malaysia [12]. 

## 2. Materials and Methods

### 2.1. Materials Used

Information on materials, reagents, and equipment used is available in Supplementary Table S1.

### 2.2. Sample Selection 

For the functional characterization of *PkAMA-1* from Peninsular Malaysia and Malaysian Borneo, clinical isolates were selected based on the largest amino acid difference between the predominant haplotypes [*PkAMA-1* domain II (*PkAMA-1*-DII)] representing respective areas under study. Hence, isolates PHG30007 (GenBank accession number MW484869) and SRWK0055 (GenBank accession number ON981708) were selected to represent Peninsular Malaysia and Malaysian Borneo, respectively.

### 2.3. Construction of Recombinant Plasmids

*Plasmodium knowlesi* DNA was extracted from the blood samples using a commercial blood extraction kit based on protocols provided by the manufacturer. The following primers were designed for *PkAMA-1*-DII PCR: a forward primer of *PkAMA-1*-DII-F: 5′ GGATCCCGTAAAAATTTAGGAAACGCA 3′ and a reverse primer of *PkAMA-1*-DII-R: 5′ GGATCCTTATTCATTGTCTACTTCTTGAGG 3′. Primers were designed specifically based on the *PkAMA-1* nucleotide sequence of isolates PHG30007 and SRWK0055. *Bam*H1 restriction sites were added to the forward and reverse primers to facilitate protein expression vector cloning. In a final volume of 25 μL, about 0.5 μg of genomic DNA was mixed with 2 mM MgCl_2_, 0.25 μM of forward and reverse primers, 0.2 mM of dNTPs, and 1 unit of *Taq* DNA polymerase buffer, as provided in the commercial PCR kit. The following steps were used to perform a single-step PCR reaction: an initial denaturation phase of 5 min at 95 °C, followed by 35 cycles of 94 °C for 1 min, 53 °C for 1 min, and 72 °C for 1 min. Finally, an elongation step at 72 °C for 10 min was carried out. Gel electrophoresis was carried out on a 1% agarose gel dyed with SYBR^®^ Safe DNA stain to visualize the PCR results.

The PCR products corresponding to isolates from Peninsular Malaysia (henceforth *PkAMA-1*-DII-P) and Malaysian Borneo (henceforth *PkAMA-1*-DII-B) were ligated into the pGEM-T^®^ TA cloning vector and transformed into competent *Escherichia coli* TOP10F’ cells. The presence of *PkAMA-1* DNA fragments in the recombinant colonies was determined using colony PCR. Positive colonies were selected, followed by plasmid extraction and DNA sequencing. BioEdit Sequence Alignment Editor was used to align the nucleotide and deduce amino acid sequences to confirm the gene integrity.

Following sequence confirmation, the recombinant plasmids of *PkAMA-1*-DII fragments were digested with the *Bam*H1 restriction enzyme and cloned into the T7 promoter-based pET-30a(+), a vector that allows expression of double polyhistidine (His)-tagged recombinant proteins in competent *E. coli* TOP10F’ cells. Directional PCR was carried out to select positive recombinant clones with the correct orientation. Positive colonies were selected, followed by plasmid extraction and sequencing. In order to enhance the recombinant protein expression using the T7 system, the plasmid was transformed into *E. coli* expression host T7 Express *lysY/I^q^*, followed by directional PCR for positive clone selection, and sequencing were repeated to confirm the integrity of the gene prior to recombinant protein expression.

### 2.4. Expression of Recombinant Proteins

*E. coli* protein expression host T7 Express *lysY/I^q^* harboring the positive recombinant plasmid of *PkAMA-1*-DII-P and *PkAMA-1*-DII-B were inoculated and propagated in Luria-Bertani (LB) broth containing 34 μg/mL of kanamycin and 35 µg/mL of chloramphenicol at 37 °C overnight. The culture was diluted to 0.1 optical density (OD) at 600 nm wavelength (OD_600_) and allowed to grow until 0.4–0.6 OD_600_. Subsequently, the bacteria culture was induced with 1 mM isopropyl β-D-1-thiogalactopyranoside (IPTG) and allowed to grow at 37 °C for 6 h. The cells were then pelleted by centrifugation at 4200 g for 10 min for harvesting.

### 2.5. Recombinant Protein Purification 

A hybrid purification method was adapted from the manufacturer’s protocol. Briefly, the expressed recombinant proteins were purified with ProBond^TM^ purification system using nickel-NTA agarose resin. Pelleted cells from the recombinant protein-producing cultures were resuspended in denaturing condition with guanidinium lysis buffer [pH 7.8, containing guanidine hydrochloride (GuHCl), monosodium phosphate (NaH_2_PO_4_), disodium phosphate (Na_2_HPO_4_), sodium chloride (NaCl)]. The protein lysates were sonicated up to the amplitude of 75% (15 sec pulse, 30 sec rest) in ice. Following centrifugation at 3000 g for 15 min at 4 °C, supernatants were collected as lysate products. The lysates were facilitated to bind with nickel-NTA agarose resins by vigorous shaking on ice for two hours. The resins were washed twice with denaturing binding buffer (pH 7.8, containing urea, NaH_2_PO_4_, Na_2_HPO_4_, NaCl), followed by two washing steps with denaturing wash buffer (pH 6.0, containing urea, NaH_2_PO_4_, Na_2_HPO_4_, NaCl). This was followed by renaturation of the proteins via four times of washings with native wash buffer (pH 8.0, containing imidazole, NaH_2_PO_4_, NaCl). Finally, the purified recombinant proteins were eluted with 5 mL of native elution buffer (pH 8.0, containing imidazole, NaH_2_PO_4_, NaCl). The recombinant protein concentration was quantified using the Bradford Assay kit. The crude and purified recombinant PkAMA-1-DII-P and PkAMA-1-DII-B proteins were visualized with sodium dodecyl sulfate-polyacrylamide gel electrophoresis (SDS-PAGE) and Western Blot assay using His-Tag monoclonal antibody. The recombinant proteins identities were also determined by matrix-assisted laser desorption ionization-time of flight mass spectrometry (MALDI-TOF MS). The dialysis of purified recombinant proteins against 1X phosphate-buffered saline (PBS) was performed for up to 18 h with three buffer changes during the dialysis at 4 °C.

### 2.6. Protein Exposure to Animals 

All experimental animals used in the present study were certified as specific-pathogen-free. All animal handling procedures were conducted in the Animal Experimental Unit, Faculty of Medicine, UM. Three experiment groups were assigned, namely the PkAMA-1-DII-P, PkAMA-1-DII-B, and the non-recombinant pET-30a(+) (negative control) groups. 

New Zealand white rabbits (one rabbit per group, 16 weeks old) were used for the experiments. Multiple administration regime was employed. For the first administration, 100 µg of purified PkAMA-1-DII-P and PkAMA-1-DII-B with complete Freund’s adjuvant (1:1 volume ratio) were injected intramuscularly into the rabbits, whereas for the subsequent boosters on weeks 4, 8, and 12 post-administration, 100 µg of purified PkAMA-1-DII-P and PkAMA-1-DII-B with incomplete Freund’s adjuvant (1:1 volume ratio) were used. Rabbit blood was collected through cardiac puncture on the 14th-week post-administration.

### 2.7. Affinity Purification of Antibody from Rabbit Serum

Polyclonal antibodies in the rabbit sera were purified by plate-based affinity purification [29]. The 96-well microplate was coated with 100 µL of coating buffer (pH 8.6, 0.05 M sodium bicarbonate) added with respective purified recombinant PkAMA-1-DII-P and PkAMA-1-DII-B (final concentration of 10 µg/mL). The coated 96-well microplate was incubated overnight at 4 °C. The next day, the plate was washed five times with 0.1% Tween-20 in 1X PBS (PBS-T) and blocked with 100 µL of 1% bovine serum albumin (BSA) in 1X PBS for one hour at 37ºC. Each well was washed with 0.1% of PBS-T five times. The rabbit sera were added (100 µL/well) and were incubated for 2 h at room temperature, with gentle rocking. The plate was washed five times with 0.1% PBS-T after the incubation. The purified antibodies (anti-PkAMA-1-DII-P and anti-PkAMA-1-DII-B) were eluted with 0.2 M glycine-HCl in BSA (pH 2.2) (100 µL/well), and the plate was incubated at room temperature for 20 min with gentle rocking. Lastly, 1 M Tris-HCl (pH 9.1) (15 µL/well) was added as neutralizing buffer. The solutions in the wells were transferred to a clean 1.5 mL centrifuge tube. The purified antibodies were stored at -20ºC. The purified antibodies were dialyzed using 1 X PBS and quantified by referring to optical density with absorbance at 280 nm.

### 2.8. Merozoite Invasion Inhibition Assay

Stage-synchronized *P. knowlesi* A1-H.1 strain culture suspension (schizont stage) was transferred into 96-well microplates with 1% parasitemia and 2% hematocrit. The wells were incubated with serially diluted (0.023–3 mg/mL) anti-PkAMA-1-DII-P and anti-PkAMA-1-DII-B antibodies in 10% horse serum-enriched RPMI medium [12]. The antibody-free culture aliquot served as the control. The culture was allowed to grow for 10 h (until the parasites in control wells reached ring stage) at 37 °C under the condition of 90% N_2_, 5% CO_2_, and 5% O_2_. The parasitemia of the culture with antibodies treatment and negative control was counted to determine the inhibition rate using the formulas stated below:

$$\left(a\right)\;\mathrm{Parasitemia}=\frac{\mathrm{parasites}\;\mathrm{infected}\;\mathrm{red}\;\mathrm{blood}\;\mathrm{cells}}{\mathrm{total}\;\mathrm{number}\;\mathrm{of}\;\mathrm{red}\;\mathrm{blood}\;\mathrm{cells}}\;\times \;100\%$$

$$\begin{array}{l} \left(b\right)\;\mathrm{Inhibition}\;\mathrm{rate}\\ =\frac{\mathrm{parasitemia}\;\mathrm{of}\;\mathrm{negative}\;\mathrm{control}-\mathrm{parasitemia}\;\mathrm{of}\;\mathrm{antibodies}\;\mathrm{treated}\;\mathrm{culture}}{\mathrm{parasitemia}\;\mathrm{of}\;\mathrm{negative}\;\mathrm{control}}\;\times \;100\% \end{array}$$

Experiments were repeated with parasites derived from five different batches of cultures as biological replicates. 

### 2.9. Statistical Analysis

GraphPad Prism 9.0 was used for data analyses using workflow summarized in Supplementary Figure S1. Briefly, data normality was evaluated using the Shapiro-Wilk normality test. For the comparison of multiple groups of data, One-way ANOVA with Tukey’s multiple comparison test (for normally distributed data) and Kruskal–Wallis test with Dunn’s post-test (for non-normally distributed data) were used. For two-group data comparison, an unpaired *t*-test (for normally distributed data) and Mann–Whitney test (for non-normally distributed data) were used. *p* values < 0.05 were interpreted as statistically significant.

## 3. Results

### 3.1. Recombinant Proteins Expression of PkAMA-1-DII-P and PkAMA-1-DII-B

**Figure 1:**
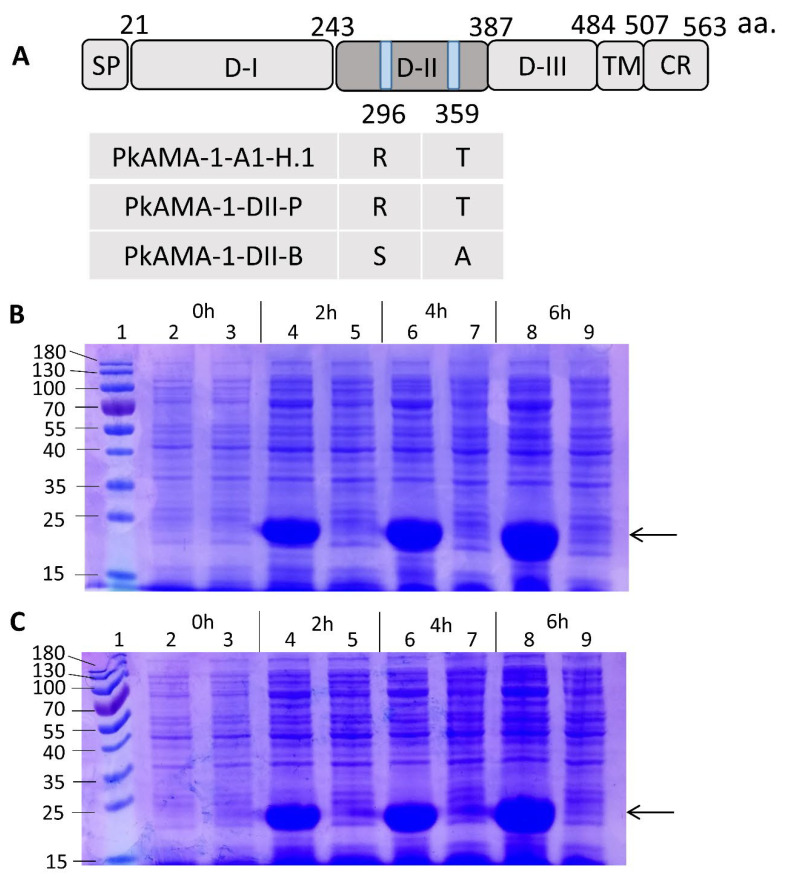

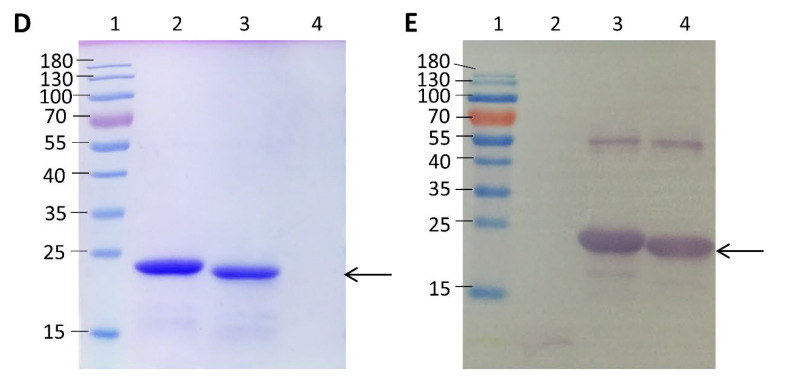
Expression of recombinant *Plasmodium knowlesi* apical membrane antigen 1-domain II (PkAMA-1-DII). (**A**) Schematic structure of PkAMA-1, which consists of signal peptide (SP), ectodomain [including domain I (D-I), domain II (D-II), and domain III (D-III)], transmembrane region (TM) and cytoplasmic region (CR). The amino acid differences between PkAMA-1-A1-H.1, PkAMA-1-DII-P and PkAMA-1-DII-B were highlighted (positions 296 and 359). (**B**) Visualisation of the Malaysian Borneo PkAMA-1-DII (PkAMA-1-DII-B) expression via SDS-PAGE. (**C**) Visualisation of the Peninsular Malaysia PkAMA-1-DII (PkAMA-1-DII-P) expression via SDS-PAGE. In both (**B**) and (**C**), lane 1: protein marker (10–180 kDa); lanes 2, 4, 6, and 8: PkAMA-1-DII protein expression at 0, 2, 4, and 6 h after IPTG induction, respectively; lanes 3, 5, 7, and 9: non-recombinant pET-30a(+) at 0, 2, 4 and 6 h after IPTG induction respectively. The PkAMA-1-DII with a size of ~21 kDa was detected after 2 h of IPTG induction (arrow). (**D**) Demonstration of purified dialyzed PkAMA-1-DII protein expression via SDS-PAGE. Lane 1: protein marker (10–180 kDa); lane 2: PkAMA-1-DII-B; lane 3: PkAMA-1-DII-P; lane 4: non-recombinant pET-30a(+). (**E**) Western Blot revealing the dialyzed purified recombinant PkAMA-1-DII expression. Lane 1: protein marker (10–180 kDa); lane 2: non-recombinant pET-30a(+); lane 3: PkAMA-1-DII-B; lane 4: PkAMA-1-DII-P. The PkAMA-1-DII-B and PkAMA-1-DII-P, with a size of ~21 kDa, were detected in both SDS-PAGE gel and Western blot (arrows).

The PkAMA-1-DII of Peninsular Malaysia and Malaysian Borneo demonstrated consistent amino acid differences at positions 296 [arginine (R) for Peninsular parasites and serine (S) for parasites from Borneo, respectively] and 359 [threonine (T) for Peninsular parasites and alanine (A) for parasites from Borneo, respectively]. Of note, the amino acid sequences of PkAMA-1 domain II derived from *P. knowlesi* A1-H.1 (reference strain; GenBank accession number LT727656) were 100% identical to those of PkAMA-1-DII-P (Figure 1A). *PkAMA-1-DII-P* and *PkAMA-1-DII-B* were successfully cloned (Supplementary Figure S2A–E). Following this, recombinant proteins of PkAMA-1-DII-P (145 amino acids) (Figure 1B) and PkAMA-1-DII-B (145 amino acids) (Figure 1C) were expressed and purified (Figure 1D). Their identity was verified with Western blot (Figure 1E) and MALDI-TOF (Supplementary Figures S3 and S4).

### 3.2. Merozoite Invasion Inhibition Assay

**Figure 2:**
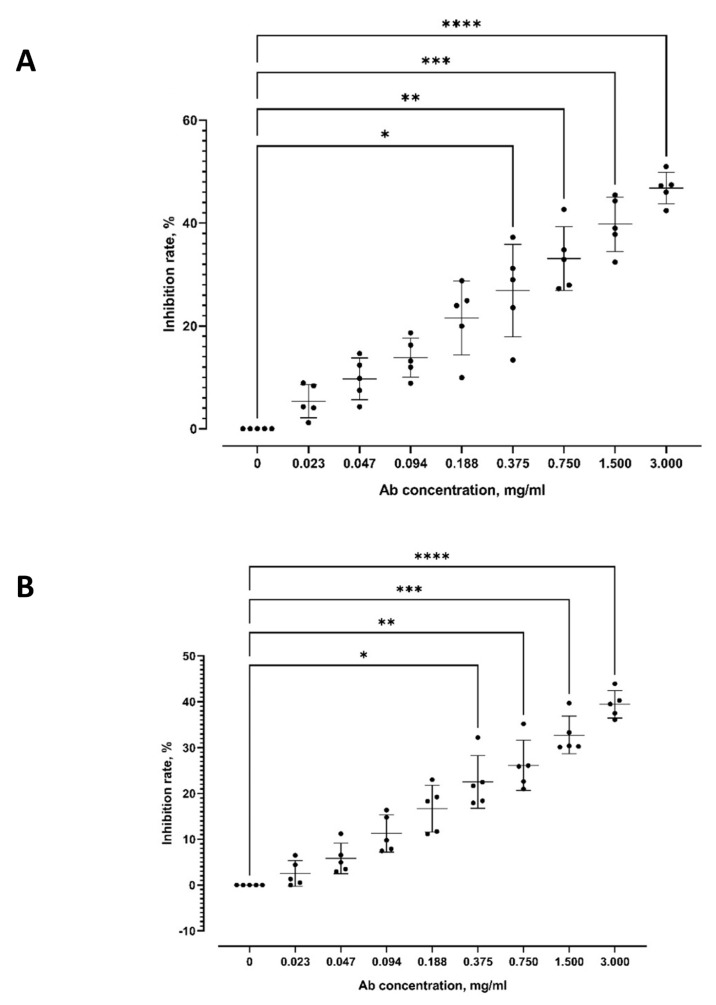

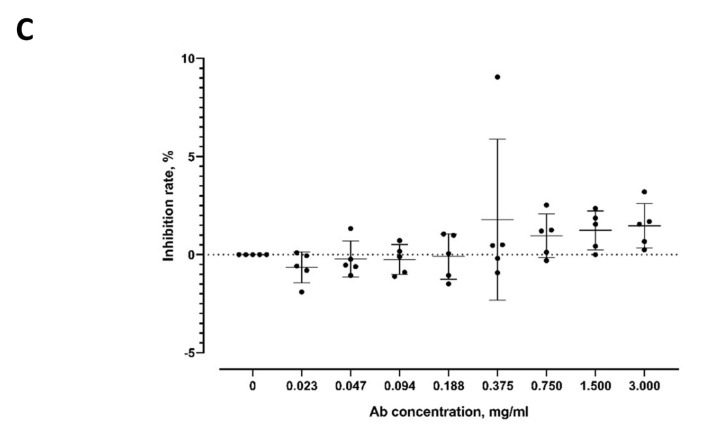
Merozoite invasion inhibition rate of gradient concentration of anti-PkAMA-1 by microscopy examination. This assay was performed in 5 biological replicates; error bars represent the means and standard deviation. (**A**) Via Kruskal-Wallis with Dunn’s post-test, significant merozoite invasion inhibition by anti-PkAMA-1-DII-P was found at 0.375 mg/mL (*p* = 0.0225), 0.75 mg/mL (*p* = 0.0035), 1.5 mg/mL (*p* = 0.0003), and 3 mg/mL (*p* < 0.0001). No significant merozoite invasion inhibition by anti-PkAMA-1-DII-P [0.023 mg/mL (*p* > 0.9999), 0.047 mg/mL (*p* > 0.9999), 0.094 mg/mL (*p* = 0.5968), 0.188 mg/mL (*p* = 0.1046)]. (**B**) Merozoite invasion inhibition by antibodies raised from PkAMA-1-DII-B-exposed rabbit. Significant merozoite invasion inhibition by antibodies raised from PkAMA-1-DII-B-exposed rabbit was found at 0.375 mg/mL (*p* = 0.0177), 0.75 mg/mL (*p* = 0.0035), 1.5 mg/mL (*p* = 0.0003), and 3 mg/mL (*p* < 0.0001). No significant merozoite invasion inhibition by anti-PkAMA-1-DII-B [0.023 mg/mL (*p* > 0.9999), 0.047 mg/mL (*p* > 0.9999), 0.094 mg/mL (*p* = 0.5367), 0.188 mg/mL (*p* = 0.0978)]. (**C**) Merozoite invasion inhibition by antibodies from the empty recombinant vector (control)-exposed rabbit. No significant merozoite invasion inhibition by antibodies from the empty recombinant vector (control)-exposed rabbit [0.023 mg/mL (*p* > 0.9999), 0.047 mg/mL (*p* > 0.9999), 0.094 mg/mL (*p* > 0.9999), 0.188 mg/mL (*p* > 0.9999), 0.375 mg/mL (*p* > 0.9999), 0.75 mg/mL (*p* > 0.9999), 1.5 mg/mL (*p* = 0.6782), 3 mg/mL (*p* = 0.3633)]. (* *p* < 0.05, ** *p* < 0.01, *** *p* < 0.001, **** *p* < 0.0001).

Antibodies against PkAMA-1-DII-P (IgG purity assessment: OD 260:280 ratio being 0.62) and PkAMA-1-DII-B (IgG purity assessment: OD 260:280 ratio being 0.62) were raised in rabbits (Supplementary Table S2). Anti-PkAMA-1-DII-P significantly inhibited *P. knowlesi* A1-H.1 from invading erythrocytes, and the inhibition rate increased with the working concentrations of the antibody (Figure 2A). Anti-PkAMA-1-DII-B demonstrated a similar trend of inhibition (Figure 2B). For anti-PkAMA-1-DII-P, significant inhibition was recorded at 0.375 mg/mL (inhibition rate: 26.87 ± 8.992%), 0.750 mg/mL (inhibition rate: 33.11 ± 6.226%), 1.500 mg/mL (inhibition rate: 39.79 ± 5.276%), and 3.000 mg/mL (inhibition rate: 46.81 ± 3.076%). For anti-PkAMA-1-DII-B, significant inhibition was recorded at 0.375 mg/mL (inhibition rate: 22.55 ± 5.768%), 0.750 mg/mL (inhibition rate: 26.17 ± 5.480%), 1.500 mg/mL (inhibition rate: 32.78 ± 4.089%), and 3.000 mg/mL (inhibition rate: 39.45 ± 3.004%). Of note, the control antibodies did not exert a significant effect on the parasite erythrocyte invasion (Figure 2C). 

**Figure 3:**
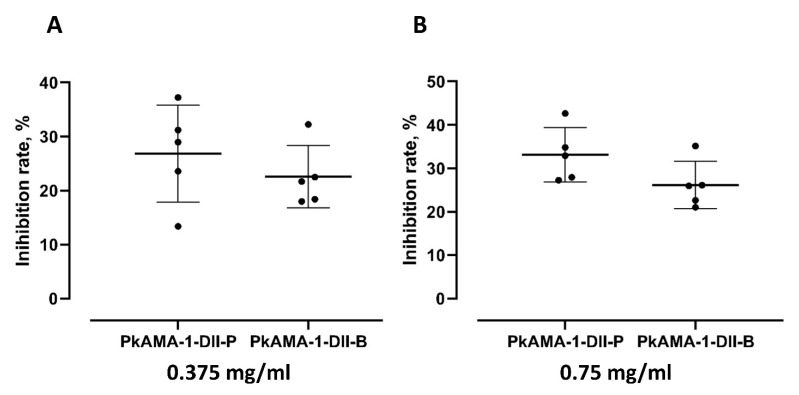

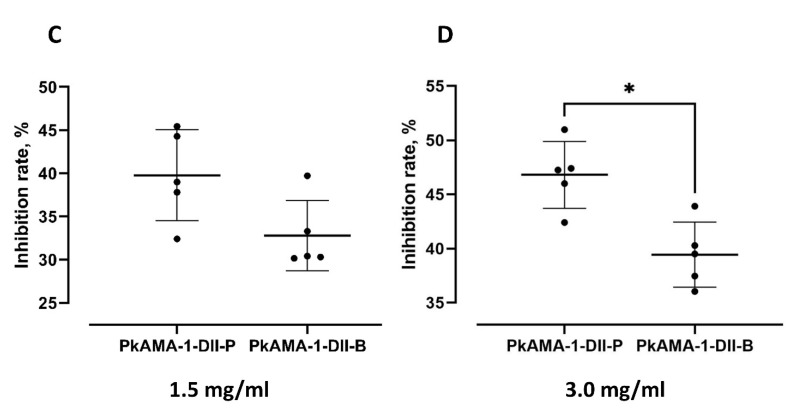
Comparison of merozoite invasion inhibition of anti-PkAMA-1-DII-P and anti-PkAMA-1-DII-B at different concentrations. (**A**) 0.375 mg/mL (*p* = 0.4206), (**B**) 0.75 mg/mL (*p* = 0.0952), and (**C**) 1.5 mg/mL (*p* = 0.0952). (**D**) A significant difference in inhibition was found between both antibodies at 3.0 mg/mL (*p* = 0.0159). (* *p* < 0.05).

We compared the parasite invasion inhibition exerted by anti-PkAMA-1-DII-P and anti-PkAMA-1-DII-B antibodies. Among the antibody concentration points that exerted significant parasite merozoite invasion (0.375, 0.75, 1.5, 3.0 mg/mL), a significant difference was found at 3.0 mg/mL, where anti-PkAMA-1-DII-P antibody exerted higher inhibition on the erythrocyte invasion (mean inhibition rate of 46.81%) than anti-PkAMA-1-DII-B (mean inhibition rate of 39.45%) (Figure 3A–D). Of the five biological replicates conducted, anti-PkAMA-1-DII-P recorded 42.41% and 50.97% as the lowest and highest invasion inhibition rate, respectively. On the other hand, the highest and lowest inhibition recorded in experiments conducted with anti-PkAMA-1-DII-B was 36.06% and 43.92%, respectively.

## 4. Discussion

In this study, we demonstrated how a minor amino acid difference in a key merozoite invasion-related protein derived from different geographical areas (Figure 1A) could give rise to antibodies that imparted different levels of invasion inhibition on *P. knowlesi* A1-H.1 [28], a reference strain derived from a clinical isolate from Peninsular Malaysia [12]. Hence, it was not surprising to see a much higher invasion inhibition on *P. knowlesi* A1-H.1 by anti-PkAMA-1-DII-P (which shared the same amino acid sequences with *P. knowlesi* A1-H.1) than by anti-PkAMA-1-DII-B. Interestingly, Muh et al. raised antibodies with recombinant PkAMA-1 constructed based on the *PkAMA-1* gene of *P. knowlesi* A1-H.1 and reported slightly higher invasion inhibition on *P. knowlesi* A1-H.1 (inhibition rate of ~50% at an antibody concentration of 1.5 mg/mL), as compared to our findings (inhibition rate of ~40% by anti-PkAMA-1-DII-P and ~30% by anti-PkAMA-1-DII-B at a working concentration of 1.5 mg/mL) [24]. Of note, the antibodies generated by Muh et al. covered domains I and II of PkAMA-1, whereas our antibodies were raised against domain II of PkAMA-1 only. Hence, it is likely that the domain II of PkAMA-1 plays a critical role in merozoite invasion, which is in parallel with earlier findings on *P. falciparum*, *P. vivax*, and *P. knowlesi* [25,30,31]. Indeed, the AMA-1 amino acid sequences of these malaria parasites are relatively conserved (Supplementary Figure S5). Previously, domain II of AMA-1 was deduced to be the domain that interacts with the rhoptry neck protein (RON 2) of the parasite during erythrocyte invasion [20]. Based on the simian W1 reference strain of *P. knowlesi*, this domain was reported to have no polymorphic sites [25]. Nevertheless, our clinical sample-based approach found this small but important variation in PkAMA-1 domain II. This suggests that the adaptation to human hosts has differentially driven the evolutionary biology of this invasion-associated protein.

The antibodies produced in our study could only exert partial invasion inhibition on the parasites, even at a high antibody concentration of 3 mg/mL. Indeed, the antibodies raised against AMA-1 of *P. knowlesi* A1-H.1 could not impart absolute merozoite invasion inhibition on *P. knowlesi* A1-H.1 as well [24]. Our findings, along with evidence contributed by previous studies, strongly suggest an alternative merozoite invasion pathway by *P. knowlesi*. The blood stages of *P. falciparum* and *P. vivax* have been demonstrated to have multiple merozoite invasion pathways [32,33]. Although the infection history between *P. knowlesi* and humans is relatively short compared to *P. falciparum* and *P. vivax*, it is still possible for this zoonotic parasite to adopt multiple invasion pathways in its relatively new intermediate host. Protein candidates such as thrombospondin-related apical membrane protein (TRAMP), rhoptry-associated protein 1 (RAP1), and surface protein of altered thrombospondin repeat (SPATR) may involve in this alternative invasion pathway as these proteins have been suggested to be important in merozoite invasion by other species of malaria parasites [34,35,36]. Nevertheless, the functions of these proteins have yet to be fully elucidated. Notably, PkAMA-1, as well as other *P. knowlesi*-derived proteins that are involved in erythrocyte invasion, are likely to carry binding domains with high interaction plasticity, in other words, relatively low host specificity. This intrinsic feature serves as an important prerequisite towards cross-species host adaptation, which leads to the transformation of this simian parasite into a zoonotic pathogen.

In falciparum malaria endemic areas, partial immunity has been demonstrated in individuals who recovered from previous episodes of malaria [37]. However, our findings may propose a different picture for the transmission of knowlesi malaria. The significant difference in invasion inhibition by the antibodies raised against PkAMA-1 from Peninsular Malaysia and Malaysian Borneo suggests that individuals recovered from knowlesi malaria acquired in one geographical area may not enjoy the similar degree of protection against *P. knowlesi* infection in another location. In fact, *P. knowlesi* derived from clinical isolates in Peninsular Malaysia and Malaysian Borneo have been shown to evolve independently [38]. Since the information regarding the genetic diversity of *P. knowlesi* genetic markers such as *PkAMA-1* in areas outside Malaysia is critically lacking, there is a need to conduct genetic studies of these zoonotic parasites that originated from other countries in Southeast Asia. Nevertheless, more work is needed to decipher the complete immuno-pathobiology of knowlesi malaria in the human population, which involves not only humans and the parasites but the simian natural hosts and the anopheline vectors as well.

In agreement with earlier studies, the domain II of PkAMA-1 demonstrates an important role in *P. knowlesi* merozoite invasion. Importantly, the minor differences found in the domain II of PkAMA-1 due to geographical segregation are adequate to vary the invasion inhibitory effect of antibodies. This spatial segregation-driven allopatric divergence in the parasite’s invasion-related gene may result from independent anthropogenic changes. This suggests a more complex-than-expected host-switching adaptability of the simian parasites from different localities, which may further complicate the One Health strategy that focuses on the human-animal-environment interface for the management, control, and prevention of zoonotic malaria. Since this study was conducted with a reference strain of *P. knowlesi*, further investigations can be conducted with these antibodies with clinical isolates collected from different geographical areas, which is a logistically challenging task. Succinctly, more research attention should be given to decipher the impact of *P. knowlesi* genetic diversity on the pathobiology of knowlesi malaria transmission as a fundamental preparation for the One Health intervention of knowlesi malaria transmission.

## Data Availability

The data presented in this study are available in this article and supplementary materials.

## Supplementary Materials

The following supporting information can be downloaded at: https://www.mdpi.com/article/10.3390/tropicalmed8010056/s1, Table S1: Information on biological materials, chemicals, software, and services used in this study. Table S2: Antibodies concentration and purity measurement. Table S3. Parasitemia was obtained from all experiment groups and the negative controls (antibody-free). Figure S1: Schematic diagram of statistical analysis. Figure S2: PCR, colony PCR, and directional PCR of PkAMA-1-DII. (A) Visualization of the PCR for *PkAMA-1*-DII-P and *PkAMA-1*-DII-B via agarose gel electrophoresis. Lane 1: 1000 bp of DNA ladder; lane 2: *PkAMA-1*-DII-P; lane 3: *PkAMA-1*-DII-B; lane 4: negative control. (B) Visualization of the colony PCR for the positive selection of *PkAMA-1*-DII-P and *PkAMA-1*-DII-B with pGEM-T^®^ TA cloning vector in TOP10F’ *E. coli* cells via agarose gel electrophoresis. Lane 1: 1000 bp of DNA ladder; lane 2–6: *PkAMA-1*-DII-P; lane 7–11: *PkAMA-1*-DII-B; lane 12: negative control. (C) Visualization of the restriction enzyme digestion of *PkAMA-1*-DII-P and *PkAMA-1*-DII-B in pGEM-T^®^ TA cloning vector via agarose gel electrophoresis. Lane 1: 1000+ bp of DNA ladder; lane 2: *PkAMA-1*-DII-P; lane 3: *PkAMA-1*-DII-B; lane 4: negative control. (D) Visualization of the directional PCR for the positive selection of *PkAMA-1*-DII-P and *PkAMA-1*-DII-B with T7 promoter-based pET-30a(+) protein expression vector in TOP10F’ *E. coli* cells via agarose gel electrophoresis. Lane 1: 1000 bp of DNA ladder; lane 2–6: *PkAMA-1*-DII-P; lane 7–11: *PkAMA-1*-DII-B; lane 12: negative control. (E) Visualization of the directional PCR for the positive selection of *PkAMA-1*-DII-P and *PkAMA-1*-DII-B with T7 promoter-based pET-30a(+) protein expression vector in *E. coli* protein expression host T7 Express *lysY/I^q^* via agarose gel electrophoresis. Lane 1: 1000 bp of DNA ladder; lane 2–3: *PkAMA-1*-DII-P; lane 4–6: *PkAMA-1*-DII-B; lane 7: negative control. The *PkAMA-1*-DII-B and *PkAMA-1*-DII-P with the length of 435 bp [in (A) and (C) (PCR using PkAMA-1-DII primers)] and 675 bp [in (B), (D) and (E) (PCR using vector primers)] were detected in agarose gel (arrows). Figure S3. The MALDI-TOF analysis revealed PkAMA-1-DII-P protein identity as *P. knowlesi* apical membrane antigen 1. Figure S4. The MALDI-TOF analysis revealed PkAMA-1-DII-B protein identity as *P. knowlesi* apical membrane antigen 1. Figure S5. Schematic structures and sequences of *P. knowlesi* AMA-1, *P. vivax* AMA-1, and *P. falciparum* AMA-1. (A) Schematic structures of *P. knowlesi* AMA-1 (PkAMA-1), *P. vivax* AMA-1 (PvAMA-1), and *P. falciparum* AMA-1 (PfAMA-1), which consists of signal peptide (SP), ectodomain, transmembrane region (TM) and cytoplasmic region (CR) (analysis using the protein signature databases, InterProScan). (B) Alignment of the amino acid sequences of PkAMA-1, PvAMA-1, and PfAMA-1. The amino acids that are conserved across the three recruited species are highlighted in black, whereas the polymorphic amino acids are highlighted in grey.
