# Supplementary material for: The Impact of Geographical Variation in Plasmodium knowlesi Apical Membrane Protein 1 (PkAMA-1) on Invasion Dynamics of P. knowlesi
Source: Trop Med Infect Dis. 2023 Jan 10;8(1):56. doi: 10.3390/tropicalmed8010056 (PMC9863575; doi:10.3390/tropicalmed8010056)
Supplement: Supplementary file 1 [file tropicalmed-08-00056-s001.zip › supplementary materials tropicalmed- 1980346.pdf]

Supplementary Material

Table S1. Information on biological materials, chemicals, softwares and services used in this study.

**Reagent/Resource**	**Reference or source**	**Identifier/ Catalog Number / Notes**
**Biological materials**		
A1-H.1 (*Plasmodium knowlesi*)	Laboratory-adapted parasite strain; maintained in Universiti Malaya lab. [3]	Used for merozoite invasion inhibition assay.
**Antibodies**		
Biotin-labelled Goat Anti-mouse Antibodies	KPL Inc., USA	Cat. No. 16-18-06
His-Tag Monoclonal Antibody	Merck KGaA., Germany	Cat. No. 70796-M
**Chemicals, Enzymes and other reagents**		
30% Acrylamide/Bis Solution	Bio-Rad, USA	Cat. No. 161-0156
5-bromo-4-chloro-3-indolyl phosphate/nitro blue tetrazolium (BCIP/NBT)	Sigma, USA	Cat. No. B5655
5X Green GoTaq® Flexi Buffer	Promega, Madison, Wisconsin, USA	Cat. No. M891A
Agarose	EURx Ltd., Gdansk, Poland	Cat. No. E0301-500
AlbuMAX II™	Gibco, USA	Cat. No. 11021-045
Alkaline Phosphotase Labeled Streptavidin	KPL Inc., USA	Cat. No. 475-3000
BamH1 Restriction Enzyme	New England Biolabs, USA	Cat. No. R0136T
Bovine Serum Albumin (BSA)	Sigma, USA	Cat. No. A9418
Chloramphenicol	Sigma, USA	Cat. No. C0378
Complete Freund's Adjuvant	Sigma, USA	Cat. No. F5881
Coomassie Blue Stain	Serva, Germany	Cat. No. 17524
D-glucose	Sigma, USA	Cat. No. G7520
DNeasy® Blood & Tissue Kit	Qiagen, Hilden, Germany	Cat. No. 69506
dNTP Mix	Promega, Madison, Wisconsin, USA	Cat. No. U1511
Giemsa	Merck, USA	Cat. No. HX87730704
Histodenz	Sigma, USA	Cat. No. D2158
Horse Serum	Gibco, USA	Cat. No. 16050122
Imidazole	Sigma, USA	Cat. No. l202
Incomplete Freund's Adjuvant	Sigma, USA	Cat. No. F5506
Isopropyl β-D-1-thiogalactopyranoside (IPTG)	Molekula, France	Cat. No. 367-93-1
Kanamycin Sulfate	Sigma, USA	Cat. No. K1377
Methanol	J.T.Baker	Cat. No. 9070-68
MgCl2, 25mM	Promega, Madison, Wisconsin, USA	Cat. No. A351H

Nickel-NTA Agarose Resins	Qiagen, Hilden, Germany	Cat. No. 30230
One Shot® Top 10 F'	Invitrogen, USA	Cat. No. C3030-05
PageRuler™ Prestained Protein Ladder, 10 to 180 kDa	Thermo Scientific, USA	Cat. No. 26616
pGEM®-T Vector System 1	Promega, Madison, Wisconsin, USA	Cat. No. A3600
Phosphate Buffer Saline (PBS)	Solarbio, China	Cat. No. P1000
QIAprep® Spin Miniprep Kit	Qiagen, Hilden, Germany	Cat. No. 27106
Quick Start™ Bovine Serum Albumin (BSA) Standard Set	Bio-Rad, USA	Cat. No. 500-0207
Roswell Park Memorial Institute (RPMI) 1640 medium	Gibco, USA	Cat. No. 23400-013
Sodium Bicarbonate	Sigma, USA	Cat. No. S5761
Sodium Dodecyl Sulfate (SDS)	Amresco, USA	Cat. No. 0227
SYBR® Safe DNA gel stain	Invitrogen, Eugene, USA	Cat. No. S33102
T7 Express *lys*Y/I^q	New England Biolabs, Inc., USA	Cat. No. C3013I
T7 Promoter-based pET-30a(+)	Merck Millipore, USA	Cat. No. 70781-3
Tween-20	Promega, Madison, Wisconsin, USA	Cat. No. H5152
Urea	Sigma, USA	Cat. No. U5378
**Software**		
BioEdit Sequence Alignment Editor Version 7.2.0	BioEdit	Used for the nucleotide and amino acid sequences alignment
GraphPad Prism Version 9.0	GraphPad	Used for the statistical analysis
**Service**		
Primers Synthesis	Integrated DNA Technologies, Inc., Singapore	Used to synthesis designed primers for PCR
Sanger Sequencing	Apical Scientific Sdn. Bhd., Malaysia	Used to determine the nucleotide sequence of DNA
Matrix Assisted Laser Desorption Ionization-time of Flight Mass Spectrometry (MALDI-TOF MS)	Medical Biotechnology Laboratory, Universiti Malaya, Malaysia	Used for protein identification
**Others**		
96-well Cell Culture Plate	Biologix, Germany	Cat. No. 07-6096
Centrifuge	Eppendorf, Germany	Model 5430r
Glass slide	Sail brand, China	Cat. No. 7107
Immun-Blot PVDF Membrane	Bio-Rad, USA	Cat. No. 1620177
Minisart® Syringe Filter, Polyethersulfone (PES), Pore Size 0.22 µm	Sartorius Minisart®, Sigma-Aldrich	Cat. No. 16532
Polypropylene Columns	Qiagen, Hilden, Germany	Cat. No. 34964
Sonic Dismembrator	Fisher Scientific, USA	Model 120
Tissue Culture Flasks 25	TPP Zellkultur und Labortechnologie, Switzerland	Cat. No. 90025
Tissue Culture Flasks 75	TPP Zellkultur und Labortechnologie, Switzerland	Cat. No. 90075
VWR® Vacuum Filtration System, 500 mL, 0.2 µm PES membrane	VWR, USA	Cat. No. 514-0332

Table S2. Antibodies concentration and purity measurement.

Sample ID	Protein Concentration	Unit	260/280	Sample Type
Anti-PkAMA-1-DII-P	3.585	mg/ml	0.62	IgG
Anti-PkAMA-1-DII-B	3.507	mg/ml	0.62	IgG

Table S3. Parasitemia obtained from all experiment groups and the negative controls (antibody-free).

(a) First biological replicate of the merozoite invasion assay

	**Parasitemia Negative control: 2.63**		
**Antibodies concentration (mg/ml)**	**Anti-PkAMA-1-DII-P**	**Anti-PkAMA-1-DII-B**	**Empty vector**
3	1.42	1.57	2.58
1.5	1.64	1.84	2.58
0.75	1.91	2.04	2.59
0.375	2.28	2.16	2.61
0.188	2.37	2.34	2.59
0.09	2.40	2.43	2.60
0.046	2.52	2.54	2.59
0.02	2.52	2.63	2.62

(b) Second biological replicate of the merozoite invasion assay

	**Parasitemia Negative control: 2.76**		
**Antibodies concentration (mg/ml)**	**Anti-PkAMA-1-DII-P**	**Anti-PkAMA-1-DII-B**	**Empty vector**
3	1.45	1.55	2.67
1.5	1.54	1.66	2.69
0.75	1.58	1.79	2.69
0.375	1.73	1.87	2.51
0.188	1.97	2.12	2.76
0.09	2.25	2.31	2.76
0.046	2.36	2.45	2.77
0.02	2.53	2.58	2.76

(c) Third biological replicate of the merozoite invasion assay

	Parasitemia<br>Negative control: 2.69		
**Antibodies concentration (mg/ml)**	**Anti-PkAMA-1-DII-P**	**Anti-PkAMA-1-DII-B**	**Empty vector**
3	1.32	1.72	2.64
1.5	1.47	1.88	2.64
0.75	1.75	2.12	2.66
0.375	1.85	2.19	2.68
0.188	2.05	2.38	2.66
0.09	2.33	2.48	2.71
0.046	2.43	2.61	2.71
0.02	2.66	2.68	2.71

(d) Forth biological replicate of the merozoite invasion assay

	Parasitemia<br>Negative control: 2.70		
**Antibodies concentration (mg/ml)**	**Anti-PkAMA-1-DII-P**	**Anti-PkAMA-1-DII-B**	**Empty vector**
3	1.42	1.63	2.69
1.5	1.83	1.88	2.70
0.75	1.95	2.00	2.71
0.375	2.06	2.11	2.71
0.188	2.16	2.18	2.74
0.09	2.26	2.30	2.70
0.046	2.37	2.52	2.71
0.02	2.46	2.58	2.75

(e) Fifth biological replicate of the merozoite invasion assay

	Parasitemia<br>Negative control: 4.20		
**Antibodies concentration (mg/ml)**	**Anti-PkAMA-1-DII-P**	**Anti-PkAMA-1-DII-P**	**Empty vector**
3	2.42	2.63	4.17
1.5	2.56	2.80	4.18
0.75	2.82	3.10	4.20

0.375	2.98	3.26	4.24
0.188	3.15	3.43	4.24
0.09	3.70	3.79	4.25
0.046	3.89	3.99	4.24
0.02	4.02	4.15	4.23

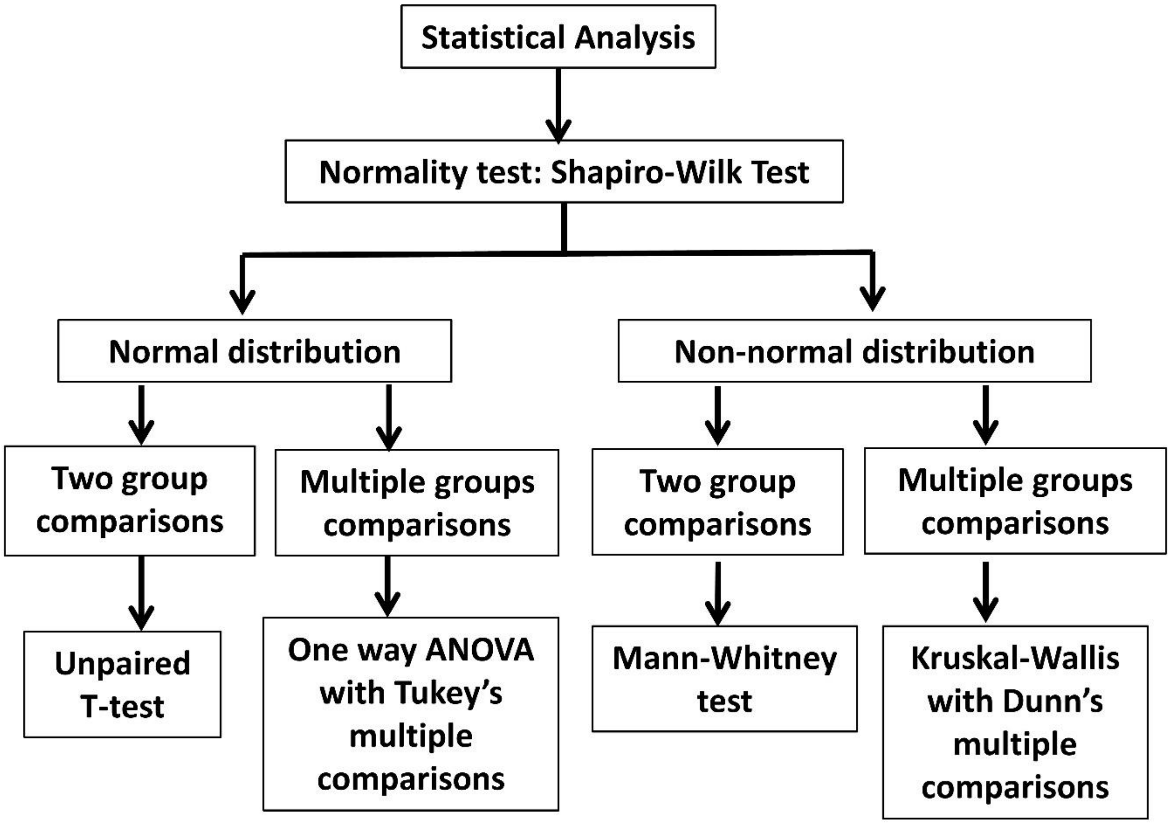

Figure S1. Schematic diagram of statistical analysis.

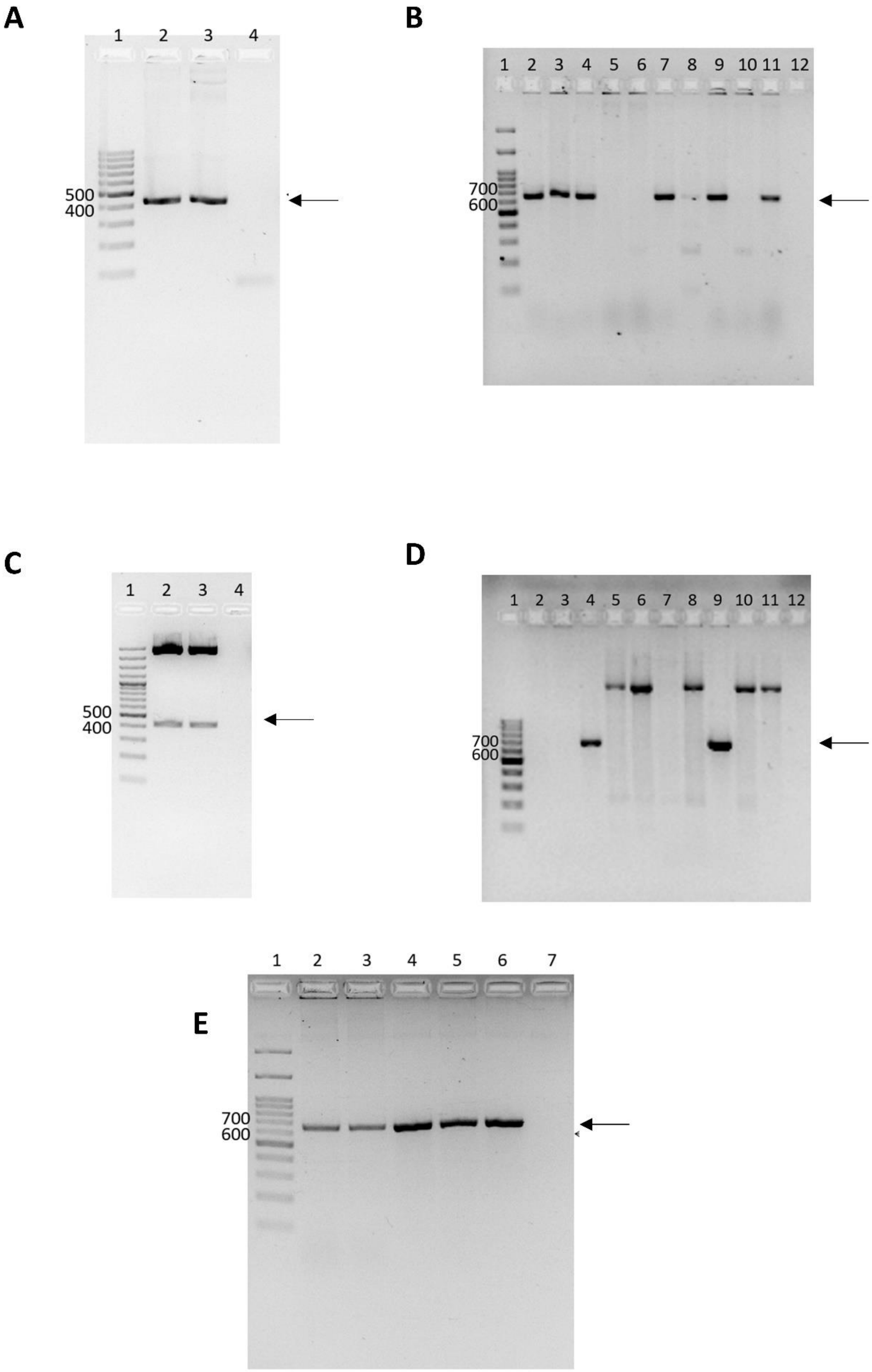
A
1 2 3 4
500
400
B
1 2 3 4 5 6 7 8 9 10 11 12
700
600
C
1 2 3 4
500
400
D
1 2 3 4 5 6 7 8 9 10 11 12
700
600
E
1 2 3 4 5 6 7
700
600

Figure S2. PCR, colony PCR and directional PCR of PkAMA-1-DII. **(A)** Visualisation of the PCR for *PkAMA-1*-DII-P and *PkAMA-1*-DII-B via agarose gel electrophoresis. Lane 1: 1000 bp of DNA ladder; lane 2: *PkAMA-1*-DII-P; lane 3: *PkAMA-1*-DII-B; lane 4: negative control. **(B)** Visualisation of the colony PCR for the positive selection of *PkAMA-1*-DII-P and *PkAMA-1*-DII-B with pGEM-T® TA cloning vector in TOP10F' *E. coli* cells via agarose gel electrophoresis. Lane 1: 1000 bp of DNA ladder; lane 2-6: *PkAMA-1*-DII-P; lane 7-11: *PkAMA-1*-DII-B; lane 12: negative control. **(C)** Visualisation of the restriction enzyme digestion of *PkAMA-1*-DII-P and *PkAMA-1*-DII-B in pGEM-T® TA cloning vector via agarose gel electrophoresis. Lane 1: 1000+ bp of DNA ladder; lane 2: *PkAMA-1*-DII-P; lane 3: *PkAMA-1*-DII-B; lane 4: negative control. **(D)** Visualisation of the directional PCR for the positive selection of *PkAMA-1*-DII-P and *PkAMA-1*-DII-B with T7 promoter-based pET-30a(+) protein expression vector in TOP10F' *E. coli* cells via agarose gel electrophoresis. Lane 1: 1000 bp of DNA ladder; lane 2-6: *PkAMA-1*-DII-P; lane 7-11: *PkAMA-1*-DII-B; lane 12: negative control. **(E)** Visualisation of the directional PCR for the positive selection of *PkAMA-1*-DII-P and *PkAMA-1*-DII-B with T7 promoter-based pET-30a(+) protein expression vector in *E. coli* protein expression host T7 Express *lysY/I*^{q} via agarose gel electrophoresis. Lane 1: 1000 bp of DNA ladder; lane 2-3: *PkAMA-1*-DII-P; lane 4-6: *PkAMA-1*-DII-B; lane 7: negative control. The *PkAMA-1*-DII-B and *PkAMA-1*-DII-P with the length of 435 bp [in **(A)** and **(C)** (PCR using PkAMA-1-DII primers)**]** and 675 bp [in **(B)**, **(D)** and **(E)** (PCR using vector primers)**]** were detected in agarose gel (arrows).

Mascot Search Results

```
User          :
Email         :
Search title  : MALDI\M2021 001\MSMS 9\B4
Database      : p_knowlesi_jan2021 knowlesi_jan2021 (16981 sequences; 12168001 residues)
Timestamp     : 25 Jan 2021 at 01:34:36 GMT
Protein hits  : A0A089Q772|A0A089Q772_PLAKN Apical membrane antigen-1 (Fragment) OS=Plasmodium knowlesi OX=5850 GN=ama-1 PE=3 SV=1
                A0A679L1Q7|A0A679L1Q7_PLAKH 6-cysteine protein OS=Plasmodium knowlesi (strain H) OX=5851 GN=PKNH_0303400 PE=4 SV=1
                A0A1Y3DJE0|A0A1Y3DJE0_PLAKN Putative Calcium/calmodulin-dependent protein kinase OS=Plasmodium knowlesi OX=5850 GN=PKNOH_S120120900 PE=4 SV=1
                A0A1Y3DLF5|A0A1Y3DLF5_PLAKN Uncharacterized protein OS=Plasmodium knowlesi OX=5850 GN=PKNOH_S120130800 PE=4 SV=1
                A0A1Y3DRL0|A0A1Y3DRL0_PLAKN Putative Kinesin-4 OS=Plasmodium knowlesi OX=5850 GN=PKNOH_S100037600 PE=3 SV=1
                A0A5K1UGJ0|A0A5K1UGJ0_PLAKH Uncharacterized protein OS=Plasmodium knowlesi (strain H) OX=5851 GN=PKNA1_C2_1257600 PE=4 SV=1
                A0A1Y3DTA8|A0A1Y3DTA8_PLAKN Uncharacterized protein OS=Plasmodium knowlesi OX=5850 GN=PKNOH_S06416200 PE=4 SV=1
                A0A1A7W1V2|A0A1A7W1V2_PLAKH Ubiquitin carboxyl-terminal hydrolase 1, putative OS=Plasmodium knowlesi (strain H) OX=5851 GN=PKNA1_H1_0209200 PE=4 SV=1
                A0A1Y3DTE4|A0A1Y3DTE4_PLAKN Putative Asparagine-rich antigen OS=Plasmodium knowlesi OX=5850 GN=PKNOH_S09517100 PE=4 SV=1
                A0A1Y3DV62|A0A1Y3DV62_PLAKN Putative Lysine decarboxylase OS=Plasmodium knowlesi OX=5850 GN=PKNOH_S02296100 PE=4 SV=1
```

Probability Based Mowse Score

Ions score is -10*Log(P), where P is the probability that the observed match is a random event.
Individual ions scores > 28 indicate identity or extensive homology (p<0.05).
Protein scores are derived from ions scores as a non-probabilistic basis for ranking protein hits.

Index

	Accession	Mass	Score	Description
1.	P21303|PK66_PLAKU	65722	562	Merozoite receptor PK66 OS=Plasmodium knowlesi (strain nuri) OX=5852 GN=PK66 PE=2 SV=2
2.	A0A0F6QH00|A0A0F6QH00_PLAKN	65627	561	Apical membrane antigen 1 OS=Plasmodium knowlesi OX=5850 GN=AMA1 PE=3 SV=1
3.	A0A089Q790|A0A089Q790_PLAKN	64644	555	Apical membrane antigen-1 (Fragment) OS=Plasmodium knowlesi OX=5850 GN=ama-1 PE=3 SV=1
4.	A0A0K0PKH1|A0A0K0PKH1_PLAKN	65746	554	Apical membrane antigen OS=Plasmodium knowlesi OX=5850 GN=ama1 PE=3 SV=1
5.	Q964I3|Q964I3_PLAKN	65736	553	Apical membrane antigen 1 OS=Plasmodium knowlesi OX=5850 GN=AMA1 PE=3 SV=1
6.	A0A5K1VGF3|A0A5K1VGF3_PLAKH	65736	553	Apical membrane antigen 1 OS=Plasmodium knowlesi (strain H) OX=5851 GN=PKNA1_C2_0931500 PE=3 SV=1
7.	A0A384KGX8|A0A384KGX8_PLAKH	65736	553	Apical membrane antigen 1 OS=Plasmodium knowlesi (strain H) OX=5851 GN=PKNH_0931500 PE=3 SV=1
8.	A0A0F6T3S0|A0A0F6T3S0_PLAKN	65706	553	Apical membrane antigen 1 OS=Plasmodium knowlesi OX=5850 GN=AMA1 PE=3 SV=1
9.	A0A0F6T319|A0A0F6T319_PLAKN	65664	553	Apical membrane antigen 1 OS=Plasmodium knowlesi OX=5850 GN=AMA1 PE=3 SV=1
10.	A0A0F6QJR3|A0A0F6QJR3_PLAKN	65692	553	Apical membrane antigen 1 OS=Plasmodium knowlesi OX=5850 GN=AMA1 PE=3 SV=1

Figure S3. The MALDI-TOF analysis revealed PkAMA-1-DII-P protein identity as *P. knowlesi* apical membrane antigen 1.

{MATRIX SCIENCE} Mascot Search Results

User	:
Email	:
Search title	: MALDI\M2021 001\MSMS 9\B3
Database	: p_knowlesi_jan2021 knowlesi_jan2021 (16981 sequences; 12168001 residues)
Timestamp	: 25 Jan 2021 at 01:34:34 GMT
Protein hits	: A0A089Q871|A0A089Q871_PLAKN Apical membrane antigen-1 (Fragment) OS=Plasmodium knowlesi OX=5850 GN=ama-1 PE=3 SV=1
	A0A679L1Q7|A0A679L1Q7_PLAKH 6-cysteine protein OS=Plasmodium knowlesi (strain H) OX=5851 GN=PKNH_0303400 PE=4 SV=1
	A0A1Y3DW07|A0A1Y3DW07_PLAKN Uncharacterized protein OS=Plasmodium knowlesi OX=5850 GN=PKNOH_S07466800 PE=4 SV=1
	A0A1Y3DN70|A0A1Y3DN70_PLAKN Putative ES2 protein OS=Plasmodium knowlesi OX=5850 GN=PKNOH_S120127000 PE=3 SV=1
	A0A1Y3DNW0|A0A1Y3DNW0_PLAKN Putative ATP-dependent RNA helicase DDX6 OS=Plasmodium knowlesi OX=5850 GN=DOZI PE=3 SV=1
	A0A1Y3DJZ4|A0A1Y3DJZ4_PLAKN Bromo domain-containing protein OS=Plasmodium knowlesi OX=5850 GN=PKNOH_S140271700 PE=4 SV=1
	A0A1Y3DWN8|A0A1Y3DWN8_PLAKN PRESAN domain-containing protein OS=Plasmodium knowlesi OX=5850 GN=PKNOH_S05370500 PE=4 SV=1
	A0A193QXI4|A0A193QXI4_PLAKH Pre-mRNA-splicing factor SYF1, putative OS=Plasmodium knowlesi (strain H) OX=5851 GN=PKNA1_C2_1455600 PE=4 SV=1
	A0A193QQB3|A0A193QQB3_PLAKH SICAvar, type I OS=Plasmodium knowlesi (strain H) OX=5851 GN=PKNA1_C2_0940500 PE=4 SV=1
	A0A1Y3DU76|A0A1Y3DU76_PLAKN Uncharacterized protein OS=Plasmodium knowlesi OX=5850 GN=PKNOH_S030339305 PE=4 SV=1

Probability Based Mowse Score

Ions score is -10*Log(P), where P is the probability that the observed match is a random event.
Individual ions scores > 29 indicate identity or extensive homology (p<0.05).
Protein scores are derived from ions scores as a non-probabilistic basis for ranking protein hits.

Index

	Accession	Mass	Score	Description
1.	A0A089RQG1|A0A089RQG1_PLAKN	64544	335	Apical membrane antigen-1 (Fragment) OS=Plasmodium knowlesi OX=5850 GN=ama-1 PE=3 SV=1
2.	A0A089Q881|A0A089Q881_PLAKN	64568	335	Apical membrane antigen-1 (Fragment) OS=Plasmodium knowlesi OX=5850 GN=ama-1 PE=3 SV=1
3.	A0A089QB03|A0A089QB03_PLAKN	64592	335	Apical membrane antigen-1 (Fragment) OS=Plasmodium knowlesi OX=5850 GN=ama-1 PE=3 SV=1
4.	A0A0F6QHW5|A0A0F6QHW5_PLAKN	65648	334	Apical membrane antigen 1 OS=Plasmodium knowlesi OX=5850 GN=AMA1 PE=3 SV=1
5.	A0A0F6T3P8|A0A0F6T3P8_PLAKN	65661	334	Apical membrane antigen 1 OS=Plasmodium knowlesi OX=5850 GN=AMA1 PE=3 SV=1
6.	A0A0F6QHY3|A0A0F6QHY3_PLAKN	65648	334	Apical membrane antigen 1 OS=Plasmodium knowlesi OX=5850 GN=AMA1 PE=3 SV=1
7.	A0A0F6QIA5|A0A0F6QIA5_PLAKN	65661	334	Apical membrane antigen 1 OS=Plasmodium knowlesi OX=5850 GN=AMA1 PE=3 SV=1
8.	A0A0F6QI94|A0A0F6QI94_PLAKN	65614	334	Apical membrane antigen 1 OS=Plasmodium knowlesi OX=5850 GN=AMA1 PE=3 SV=1
9.	A0A089Q871|A0A089Q871_PLAKN	64582	334	Apical membrane antigen-1 (Fragment) OS=Plasmodium knowlesi OX=5850 GN=ama-1 PE=3 SV=1
10.	A0A089QDF8|A0A089QDF8_PLAKN	64559	334	Apical membrane antigen-1 (Fragment) OS=Plasmodium knowlesi OX=5850 GN=ama-1 PE=3 SV=1

Figure S4. The MALDI-TOF analysis revealed PkAMA-1-DII-B protein identity as *P. knowlesi* apical membrane antigen 1.

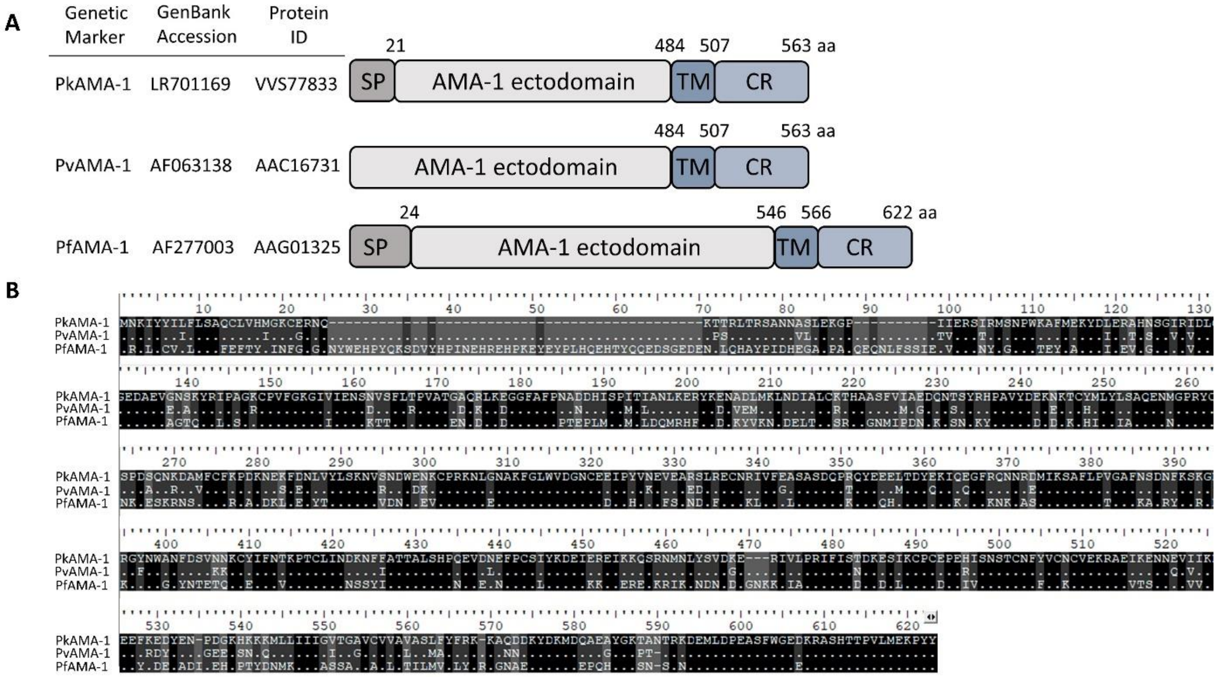

Figure S5. Schematic structures and sequences of *P. knowlesi* AMA-1, *P. vivax* AMA-1 and *P. falciparum* AMA-1. **(A)** Schematic structures of *P. knowlesi* AMA-1 (PkAMA-1), *P. vivax* AMA-1 (PvAMA-1) and *P. falciparum* AMA-1 (PfAMA-1), which consists of signal peptide (SP), ectodomain, transmembrane region (TM) and cytoplasmic region (CR) (analysis using the protein signature databases, InterProScan). **(B)** Alignment of the amino acid sequences of PkAMA-1, PvAMA-1 and PfAMA-1. The amino acids that are conserved across the three recruited species are highlighted in black, whereas the polymorphic amino acids are highlighted in grey.
