# Supplementary figures and images for: The Impact of Geographical Variation in Plasmodium knowlesi Apical Membrane Protein 1 (PkAMA-1) on Invasion Dynamics of P. knowlesi
Source: Trop Med Infect Dis. 2023 Jan 10;8(1):56. doi: 10.3390/tropicalmed8010056 (PMC9863575; doi:10.3390/tropicalmed8010056)

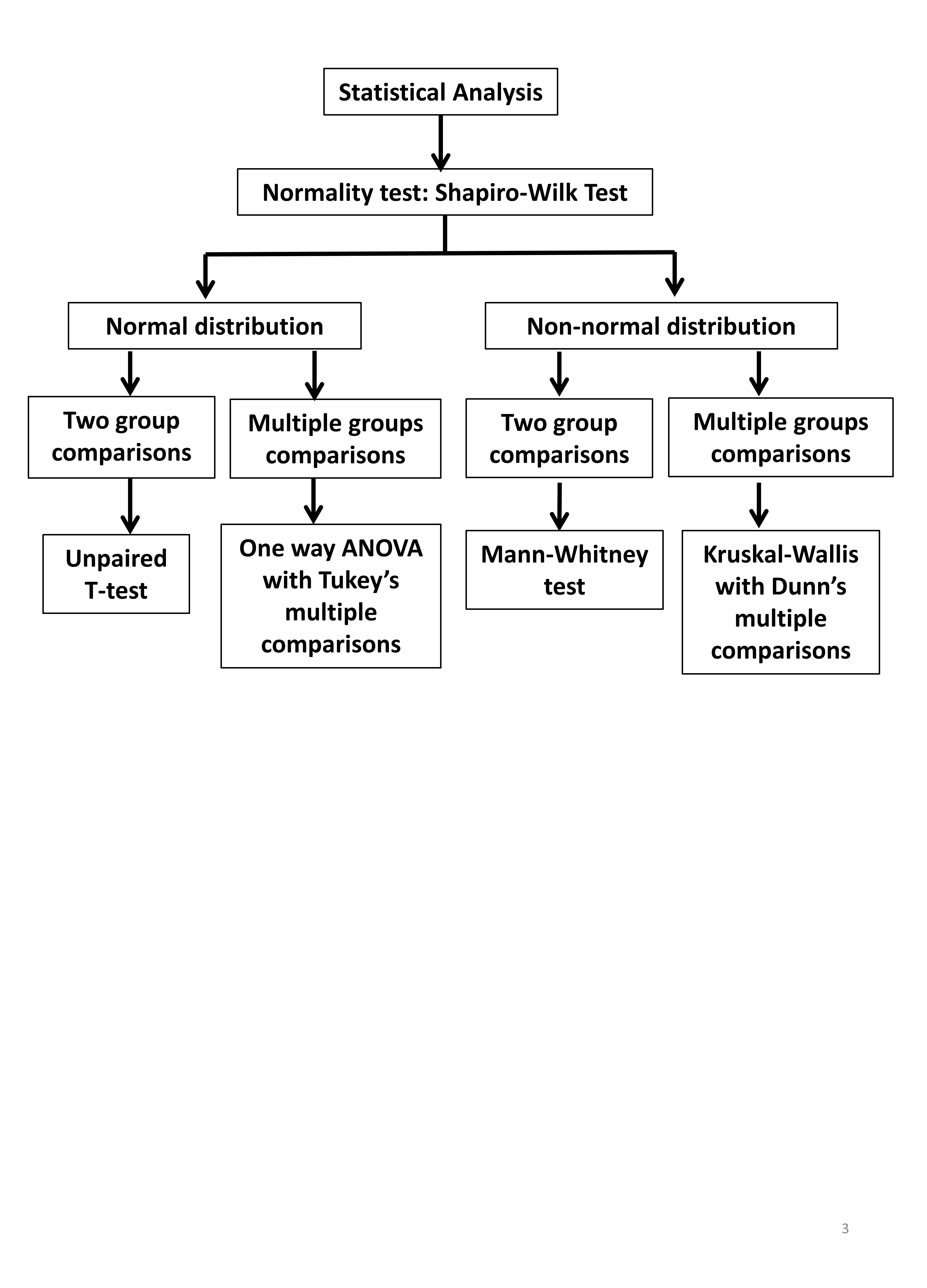

Supplement: Supplementary file 1 [file tropicalmed-08-00056-s001.zip › Figure S1.jpg]

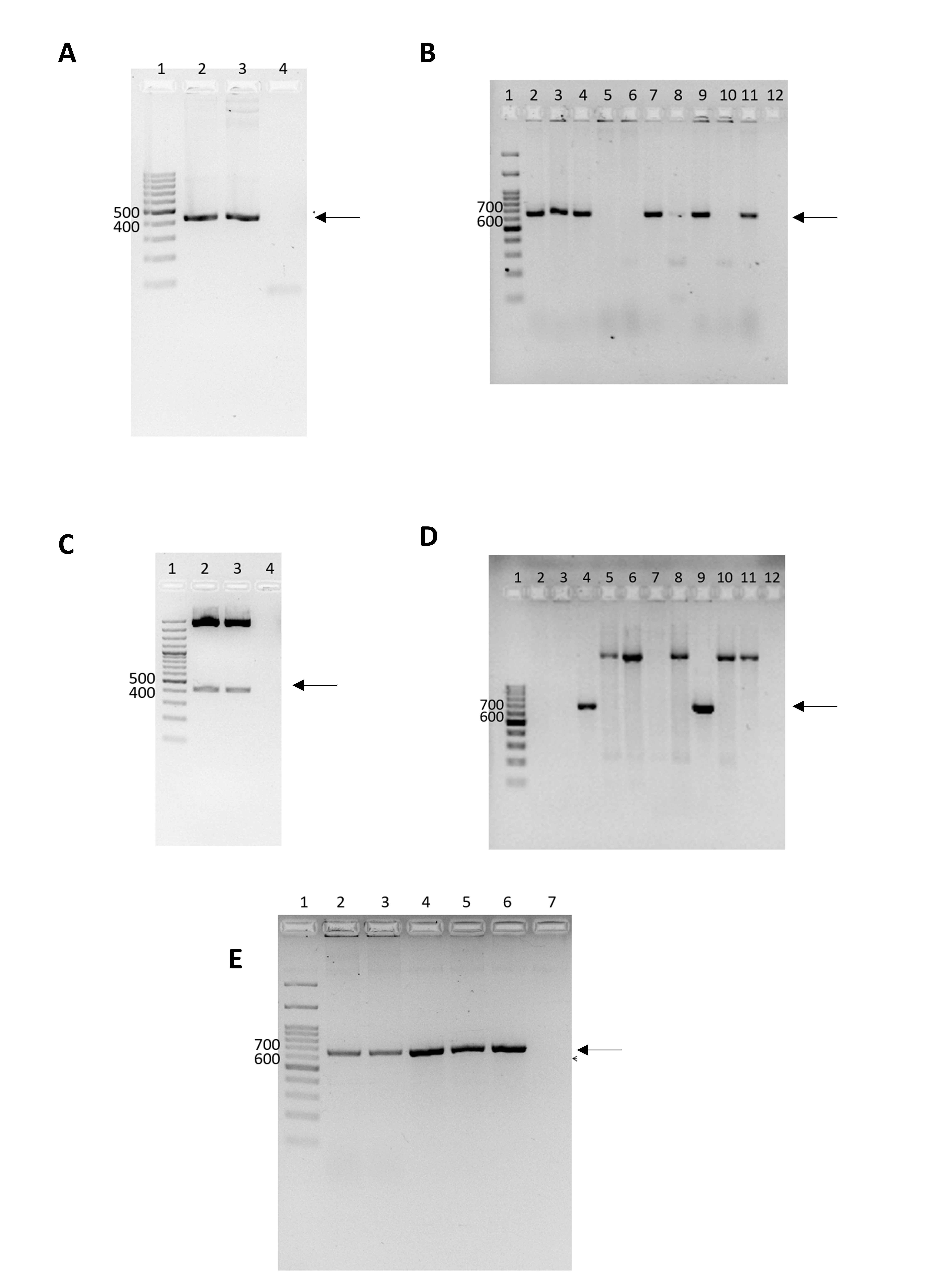

Supplement: Supplementary file 1 [file tropicalmed-08-00056-s001.zip › Figure S2.jpg]

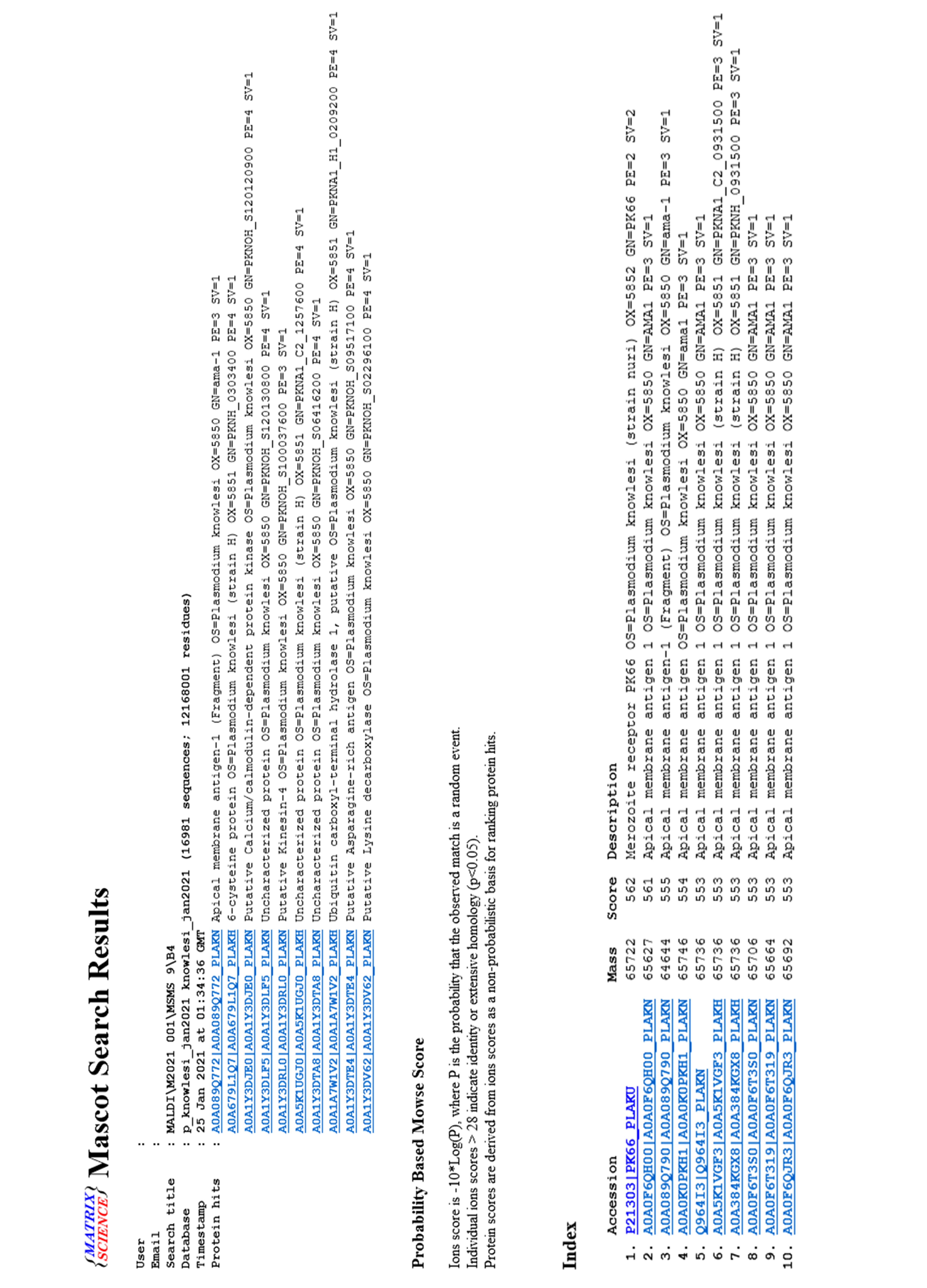

Supplement: Supplementary file 1 [file tropicalmed-08-00056-s001.zip › Figure S3.jpg]

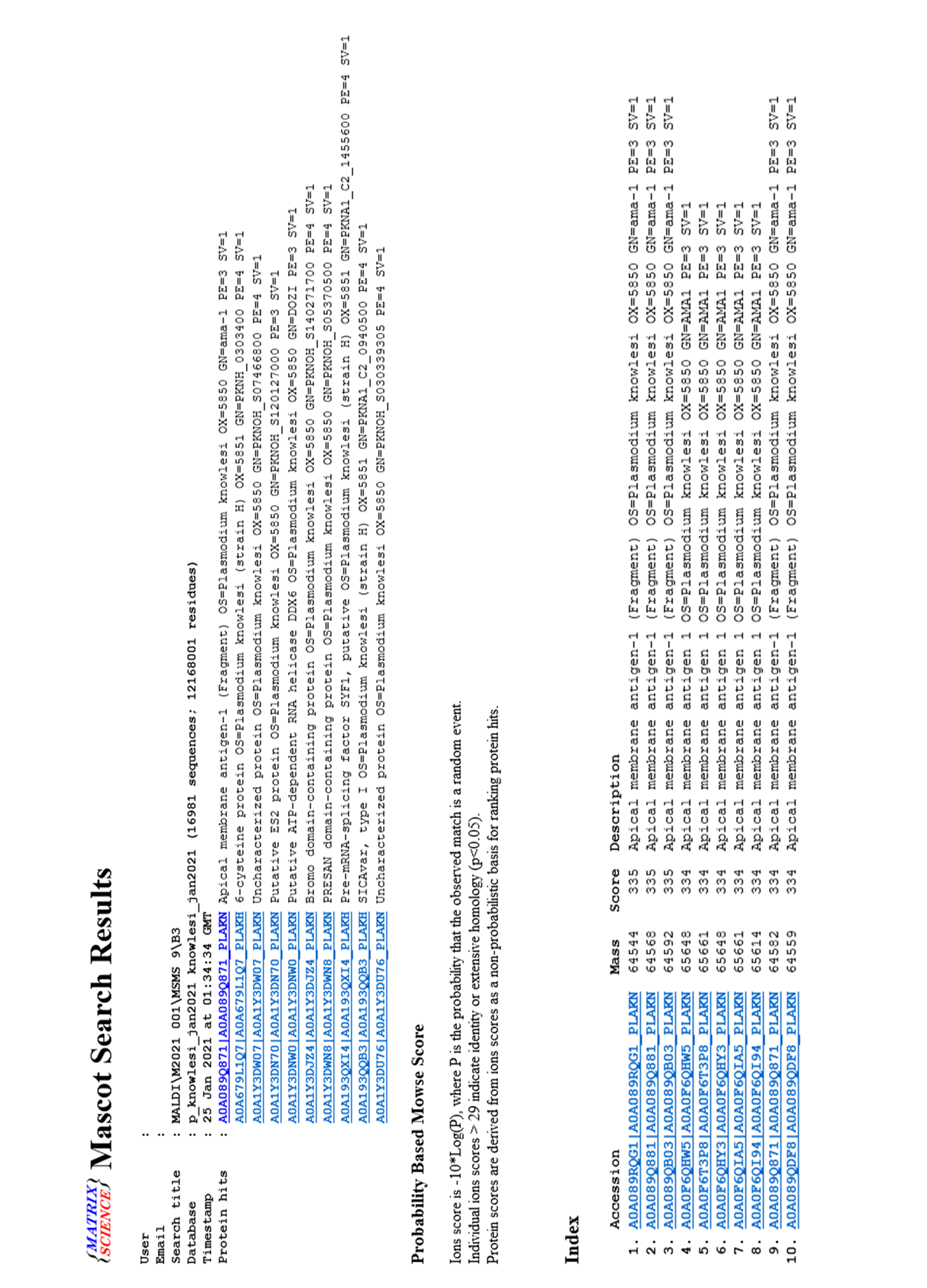

Supplement: Supplementary file 1 [file tropicalmed-08-00056-s001.zip › Figure S4.jpg]

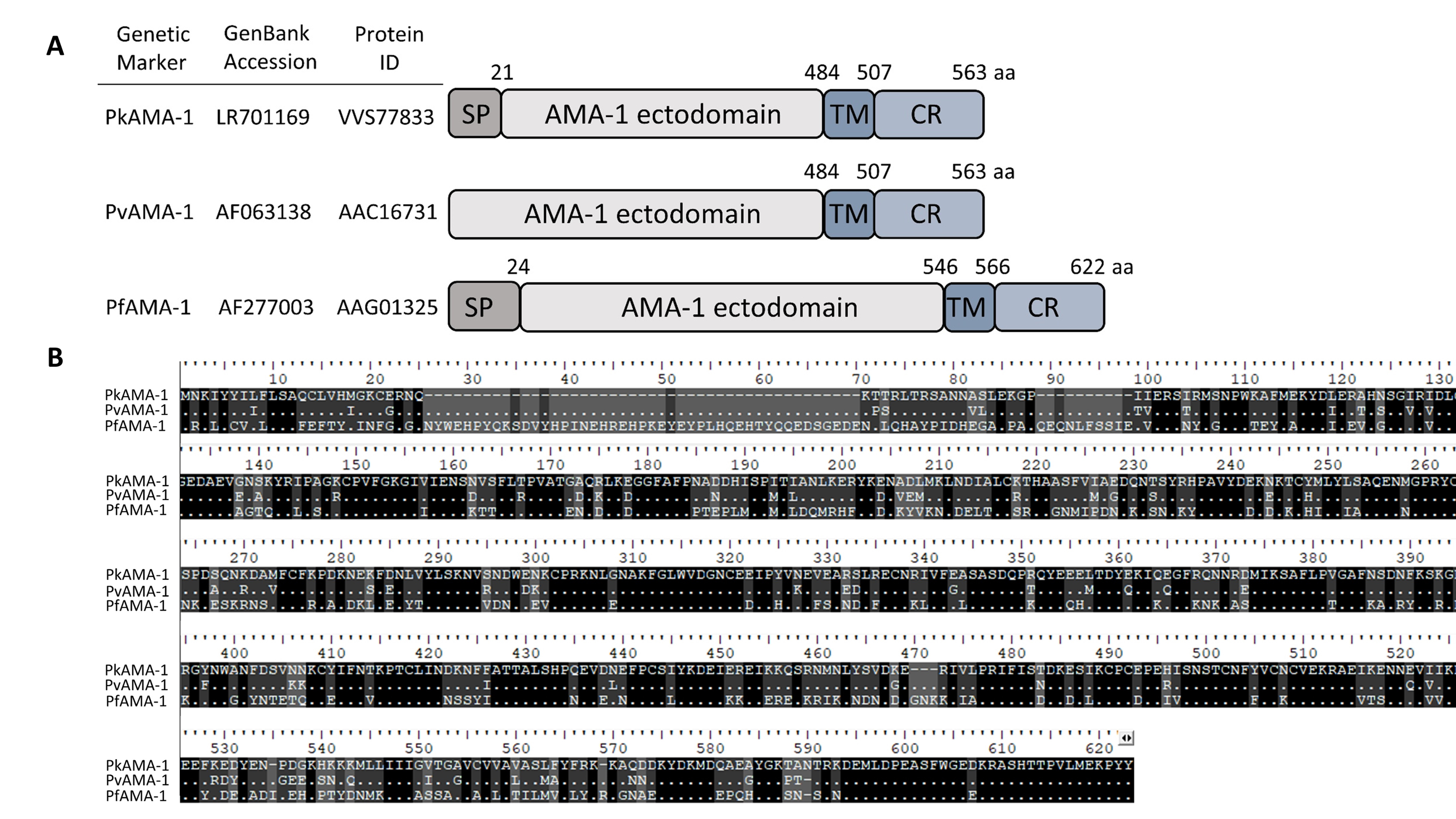

Supplement: Supplementary file 1 [file tropicalmed-08-00056-s001.zip › Figure S5.jpg]
